# Internally architectured materials with directionally asymmetric friction

**DOI:** 10.1038/srep10732

**Published:** 2015-06-04

**Authors:** Ehsan Bafekrpour, Arcady Dyskin, Elena Pasternak, Andrey Molotnikov, Yuri Estrin

**Affiliations:** 1 Centre for Advanced Hybrid Materials, Department of Materials Engineering, Monash University, Clayton, Victoria 3800, Australia; 2 School of Fashion and Textiles, RMIT University, 25 Dawson Street, Brunswick, 3056, Australia; 3 School of Civil and Resource Engineering, The University of Western Australia, 35 Stirling Highway, Crawley, WA 6009, Australia; 4 School of Mechanical and Chemical Engineering, The University of Western Australia, 35 Stirling Highway, Crawley, WA 6009, Australia; 5 Laboratory of Hybrid Nanostructured Materials, Moscow Institute of Steel and Alloys, Leninsky prosp. 4, Moscow 119049, Russia

## Abstract

Internally Architectured Materials (IAMs) that exhibit different friction forces for sliding in the opposite directions are proposed. This is achieved by translating deformation normal to the sliding plane into a tangential force in a manner that is akin to a toothbrush with inclined bristles. Friction asymmetry is attained by employing a layered material or a structure with parallel ‘ribs’ inclined to the direction of sliding. A theory of directionally asymmetric friction is presented, along with prototype IAMs designed, fabricated and tested. The friction anisotropy (the *ξ*-coefficient) is characterised by the ratio of the friction forces for two opposite directions of sliding. It is further demonstrated that IAM can possess very high levels of friction anisotropy, with *ξ* of the order of 10. Further increase in *ξ* is attained by modifying the shape of the ribs to provide them with directionally dependent bending stiffness. Prototype IAMs produced by 3D printing exhibit truly giant friction asymmetry, with *ξ* in excess of 20. A novel mechanical rectifier, which can convert oscillatory movement into unidirectional movement by virtue of directionally asymmetric friction, is proposed. Possible applications include locomotion in a constrained environment and energy harvesting from oscillatory noise and vibrations.

Friction has been the subject of innumerable studies over centuries if not millennia, with some of the first documented studies traced back to the work of Aristotle^1^. Despite the seminal contributions to our current understanding of friction by such giants as Leonardo da Vinci, Coulomb and Amontons, controlling and influencing friction at different length scales still remains a hot topic of research^2^. Frictional effects are omnipresent in everyday life; they also play a crucial role in multifarious engineering applications. Thus, friction enables both wheeled locomotion and braking. However, it is also a source of undesirable effects, such as e.g. reduced efficiency of combustion engines due to friction between piston and cylinder. A number of researchers conducted studies on directionally dependent friction where a body will readily move relative to another one it is in contact with when an external tangential force is applied in one direction, but not in the opposite direction^3^,^4^,^5^,^6^,^7^.

We would like to emphasize that in this treatise we are not considering a directional dependence of friction associated with surface anisotropy, which case was investigated elsewhere^8^,^9^. Rather, the focus is on asymmetry of friction with respect to forward vs. backward sliding along a given direction. Hereafter we will use the term *directionally asymmetric* friction in order to distinguish this phenomenon from directionally dependent friction, which implies surface anisotropy.

The effect of directionally asymmetric friction can be achieved by making the sliding body anisotropic in such a way that, in the presence of a normal constraint, it ensures coupling between the normal stress and the shear stress, τ, Fig. 1a. In other words, we consider the situations when instead of normal force normal strain (or displacement) is applied. As a result, differences in the friction force with respect to forward and backward movements are achieved through an effect on the magnitude of the resultant normal stress rather than through cohesion or the intrinsic coefficient of friction. This ensures that the effect is not dependent on the type and quality of the contacting surfaces and enables mobility in different environments. A particular way to create an anisotropy effect is by using Hawkins’ micro-machines, Fig. 1b^10^.

Directionally asymmetric friction is abundant in living nature, and is found, e.g., in the setae structure of geckos, beetles, flies, frogs, spiders and lizards feet, as recently reported in^11^,^12^,^13^,^14^,^15^,^16^,^17^,^18^,^19^. Such asymmetric friction behaviour commonly stems from special surface morphology, exemplified by brush-like profiles with inclined bristles^20^,^21^,^22^,^23^,^24^,^25^,^26^. In this study, we propose a design of IAMs based on machine augmented composites (MACs) first introduced by Hawkins and co-workers^10^,^27^,^28^,^29^. MACs represent a new type of hybrid materials consisting of a matrix and micro-machines embedded in it. While the Hawkins group focused on linear machines, here we extend this research by introducing strongly non-linear micro-mechanisms.

Using finite element simulations and physical models considered in^29^, we will demonstrate that a tangential force externally applied to a body sliding on the surface of an IAM induces a normal reaction force from the IAM. This alters the resultant normal force and thus affects the frictional resistance force. Depending on the direction of sliding, the resultant resistance force tangential to the interface between the IAM and the sliding body can be either reduced or enhanced. This is a promising property, as devices based on directional asymmetry of friction have the potential to be utilized for various applications, including energy absorbers, protective gear, reinforced adhesives, sporting goods such as skis, etc. Prototype devices, in which the proposed concept was realised, were produced by the state-of-the-art 3D printing technology. Some of these IAMs will be presented below, as well as in Supplementary online.

## Results

### Directionally Asymmetric Friction

**Figure 1:**
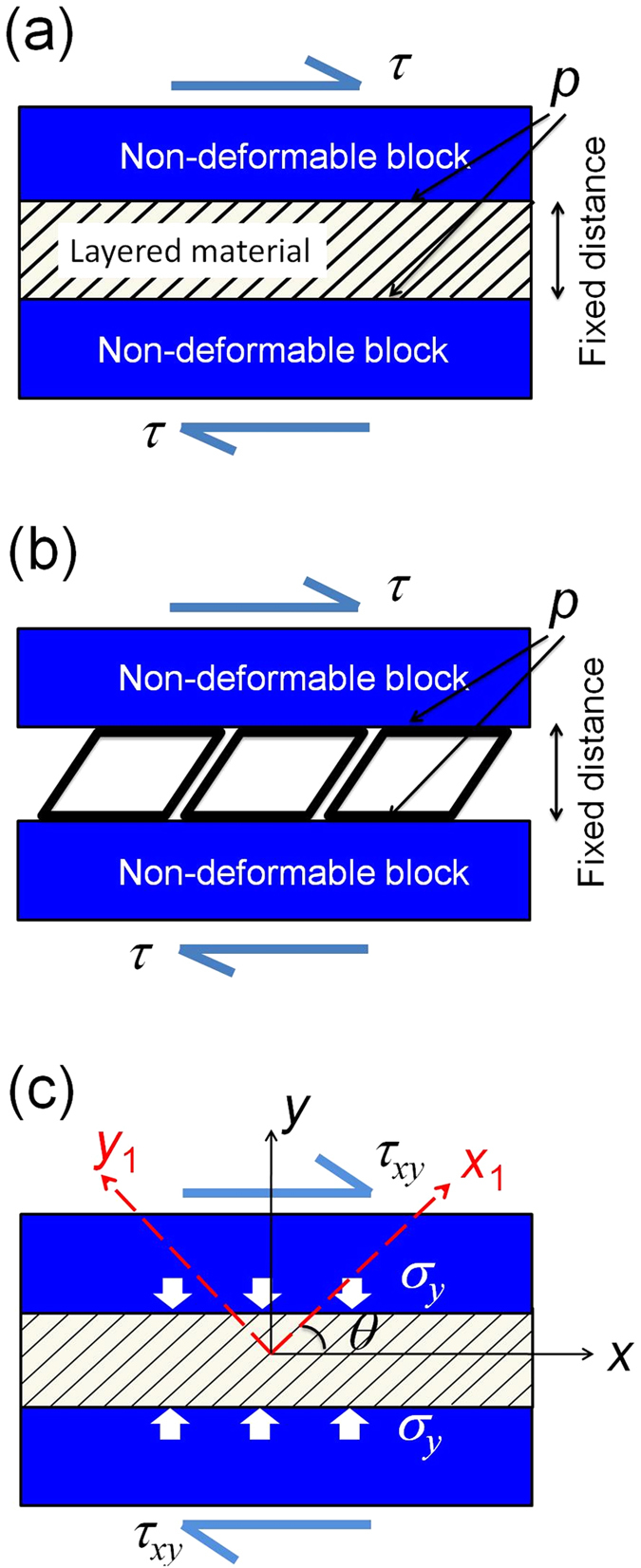
Possible realisations of directionally asymmetric friction. **a,** layered material with inclined layers (the contact pressure *p* depends upon the direction of sliding; τ denotes the applied shear stress). **b**, Hawkins’ machine augmented composite (MAC)^10^. **c,** schematics of directionally asymmetric friction, see text. We presume that the normal stress *p* or 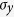 is generated by the applied normal strain and hence due to the material or structural anisotropy, it depends upon the direction of the applied shear stress.

The concept of directionally asymmetric friction characterised by different magnitudes of the friction force in the opposite sliding directions is illustrated in Fig. 1c. A slider with directionally asymmetric friction in horizontal direction is represented in this figure by a middle block constrained in normal direction by two rigid bodies shown in black. An unconstrained slider would respond by a vertical strain, ε_*y*_, which would have different values when acted upon by a shear stress τ_*xy*_=τ applied in the two opposite directions. (Obviously, a stress component τ_*yx*_ of the same magnitude is also generated by the constraint). Due to the vertical constraint, the slider cannot develop a strain ε_*y*_. Instead, a normal stress component σ_*y*_ is produced whose magnitude depends on the sign of τ_*xy*_, which is determined by the direction of motion of the slider. The role of anisotropy of the slider in this setup is to ensure direction-dependent coupling between shear stress and normal strain.

**Figure 2:**
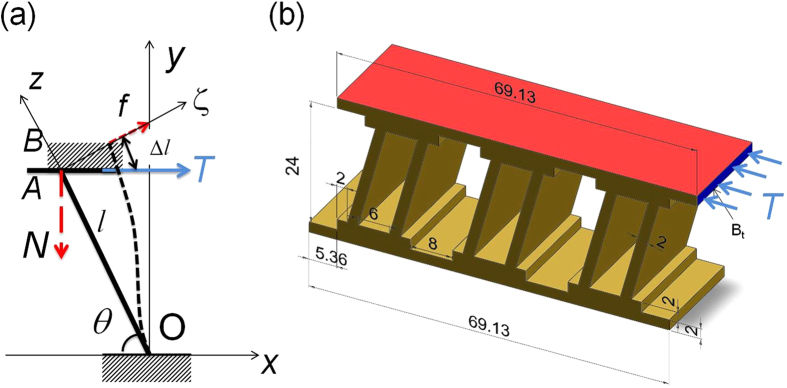
Z-machine to create asymmetric friction. **a,** Representation of the basic element of a machine realising asymmetric friction as a straight beam of length *l* clamped at point O. The other end of the beam is attached to slider *A*, which slides along a rigid block *B* under force *T*. The beam is deflected under the normal projection *f* of force *T*. If the rigid block were not there, the deflection would be as indicated by the dotted line (grossly exaggerated). The axial beam deformation in the presence of the rigid block creates a force *N*. **b,** drawing of a Z-machine with an inclination angle of 75°. The dimensions (in mm) shown in this schematic picture are those used in the actual structures produced by 3D printing and tested in this work, see below.

Conversely, if the constraint imposes upon the slider a compressive strain, −ε_*y*_^0^ (the minus sign signifying compression), the elastic response of the slider owing to this coupling will produce a shear stress whose magnitude will depend on the direction of sliding. This elastic response is expressed by a general anisotropic Hooke’s law in the following matrix form^30^:


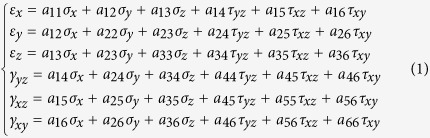


where (*a*_*ij*_) is the (symmetric) compliance matrix and γ_*ij*_=2ε_*ij*_, *i*, *j*=*x*, *y*, *z* in the Cartesian coordinate system of Fig. 2. Conventional notation for the components of the stress and the strain tensors are used.

Assuming that in our simplified case 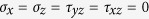 applies, one obtains:


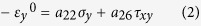


By combining equation (2) with the conventional Coulomb friction law,


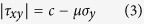


where *c* is the cohesion and μ the coefficient of friction, one obtains for a given compressive stress


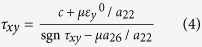


sgn x = 1 if x>0 and −1 if x<0. We now consider different cases:

Case 1: *a*_26_>0, τ_*xy*_ >0.

It follows from equation (4):


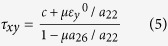


In this case the horizontal movement of the slider meets the highest resistance. Henceforth, this direction of sliding is called the *hard direction*. The friction stress has a singularity when


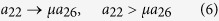


In this situation, the friction force acting against sliding in this direction is infinite. In reality this will lead to failure of the system. We also note that the case 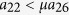 corresponds to τ_*xy*_<0, which is precluded by the assumptions of Case 1.

Case 2: *a*_26_>0, τ_*xy*_ <0.

Now equation (4) yields


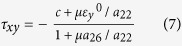


This corresponds to sliding in the direction opposite to that of Case 1, and it meets the least resistance. Henceforth this direction of sliding will be referred to as the *easy direction*. The *friction asymmetry coefficient, ξ* (defined as the ratio of the magnitudes of friction forces in the hard and easy directions) is given by


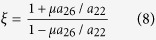


It is seen to be determined solely by the coefficient of friction and the ratio *a*_26_/*a*_22_. Notably, the friction asymmetry coefficient is independent of both the nominal normal strain and the magnitude of the cohesion.

Equation (2) shows that in the absence of an imposed strain, i.e. for ε_*y*_^0^ = 0, the ratio *a*_26_/*a*_22_ governs the degree of anisotropic coupling, as it represents the proportionality coefficient between the applied shear stress and the normal stress it induces or between the tangential force and the ensuing normal force. In the following, it will be referred to as the *force transformation coefficient,*


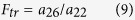


The friction asymmetry coefficient can now be expressed in terms of *F*_*tr*_ as


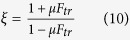


When a non-zero strain ε_*y*_^0^ is imposed, we can introduce a nominal normal stress


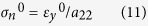


and re-write the expression for the sliding stress as


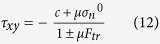


where the ‘+’ and ‘−’ signs in the denominator refer to the easy and the hard sliding direction, respectively.

Case 3 (*a*_26_<0, τ_*xy*_ <0) and Case 4 (*a*_26_<0, τ_*xy*_ >0) correspond to Cases 1 and 2, but for sliding in the respective reverse directions. Consequently, equations (4) and (8) remain the same, but refer to sliding in the directions opposite to Cases 1 and 2.

From the above considerations, particularly from equation (10), it is obvious that *F*_*tr*_ is the crucial design parameter for control of the friction asymmetry coefficient*, ξ*. It is therefore instrumental to relate the force transformation coefficient to the material properties and the inner architecture of the slider. This will enable tuning the elastic compliance coefficients *a*_26_ and *a*_22_ in the desired way, to maximize *F*_*tr*_ thus also maximizing *ξ*. The compliance coefficients *a*_26_ and *a*_22_ can readily be determined in simple cases of anisotropy, such as transverse isotropic and orthotropic ones, if the axes of anisotropy are inclined to the direction of sliding, as shown in Fig. 1c. This case is considered in the Supplementary online. Using equation (S2) obtained therein, an expression for the force transformation coefficient for a slider containing separate parallel layers, or ribs, as in a Hawkins micromachine, is derived after some simplifications:


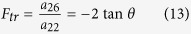


Here θ is the inclination angle of the layers or the ribs of a Hawkins Z-machine. This corresponds to the above Case 3, *a*_26_<0, τ_*xy*_ <0. According to equation (8), the maximum value of the friction asymmetry coefficient *ξ* (i.e. infinity) is reached when the equation 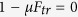 holds. Considering the negative sign of sgn *τ*_*xy*_ in Eq. (4), which underlies Eq. (8), the angle of layer inclination θ_max_ corresponding to the maximum frictional resistance is given by


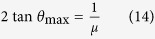


By introducing the *friction angle* φ, such that *μ*=tan φ, we obtain the following expression for θ_max_:


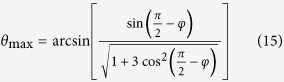


equation (15) shows the dependence of θ_max_ on the friction angle φ (see a plot of this equation in Supplementary Fig. S1 online). It is seen that the inclination angle of the layers corresponding to the maximum magnitude of the coefficient of friction decreases monotonically as the friction angle increases. The friction angle of π/2 corresponds to infinite coefficient of friction and hence cannot be reached, which represents a degenerate case of zero inclination angle θ_max_.

### An Example of Structure Exhibiting Directionally Asymmetric Friction

Hawkins *et al.*^10^,^27^,^28^ and Bafekrpour *et al.*^29^ demonstrated that MACs possess the ability to convert an applied compressive load into a lateral displacement and, vice versa, a tangential shear force into a normal displacement. As discussed in the previous section, this ability is controlled by the force transformation factor, *F*_*tr*_. We now use this concept to analyse the basic element of the IAMs shown in Fig. 2a. To this end we will calculate the force transformation coefficient, *F*_*tr*_, defined as the ratio of the normal force created by a shear force in the absence of an imposed normal displacement to this shear force. The force transformation coefficient is a characteristic particularly suitable for modelling of micromachines with separate force transfer elements. The friction asymmetry coefficient, *ξ*, is expressed in terms of *F*_*tr*_ through equation (8).

We note that the friction asymmetry coefficient is greater than unity for any values of the coefficient of friction μ and the force transformation coefficient *F*_*tr*_ and tends to infinity when *F*_*tr*_ approaches the value of 1/μ.

The basic element we consider, which mimics an individual rib of a Z-machine, is represented by a straight beam clamped at one end (point O), see Fig. 2a. The other end is attached to a slider *A*, which slides over a stationary rigid block *B* without loss of contact. No rotations are allowed. Calculating the force transformation coefficient for this basic element is straightforward, and it is presented in Supplementary online. The result is given by equation (S9), which reads.


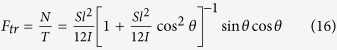


where *I* is the moment of inertia, *S* the cross-sectional area, *l* the length, and θ the inclination angle of the beam. To examine the expression for *F*_*tr*_ given by equation (16), a model of a MAC^10^,^27^,^28^ built with rigid Z-machines inclined at 75° to the horizontal plane as shown in Fig. 2b was considered. The top and bottom boundaries of the model were fixed in vertical direction, other directions were free. The force *T* was uniformly applied to boundary *B*_*t*_ (blue area) and the resulting force on the constrained red surface was measured to calculate the force transformation coefficient. Linear brick elements with 8 nodes and a size of 0.75 mm were used for meshing the model, in which centimeter-scale elements were employed. (Further reduction in the mesh size did not produce any appreciable increase in accuracy). Assuming that all machine ribs have rectangular cross-sections with side dimensions of *a* = 2 mm, *b* = 20 mm and a length *l* = 24/sin75° mm (suggested by the experimental examples considered below), and using the Poisson’s ratio of 0.1, a value of 3.4 is obtained for force transformation coefficient utilizing equation (16). The calculation was based on the assumption that the force transformation coefficient of the entire structure is the same as that of a single beam and neglecting short thick parts at the beam ends where they are attached to the platens. The finite element model presented in Fig. 2b resulted in the value of 3.35 for the force transformation coefficient, which suggests equation (16) is adequate. (The effect of Poisson’s ratio of the material of the rib on *F*_*tr*_ is presented in Supplementary Table S1 online). The comparison of numerical and analytical results also suggests that the additional material at the beam connections with the platens of the device shown in Fig. 2b has little influence on the force transformation coefficient.

### Directionally Asymmetric Friction IAMs Based on Z-machines

**Figure 3:**
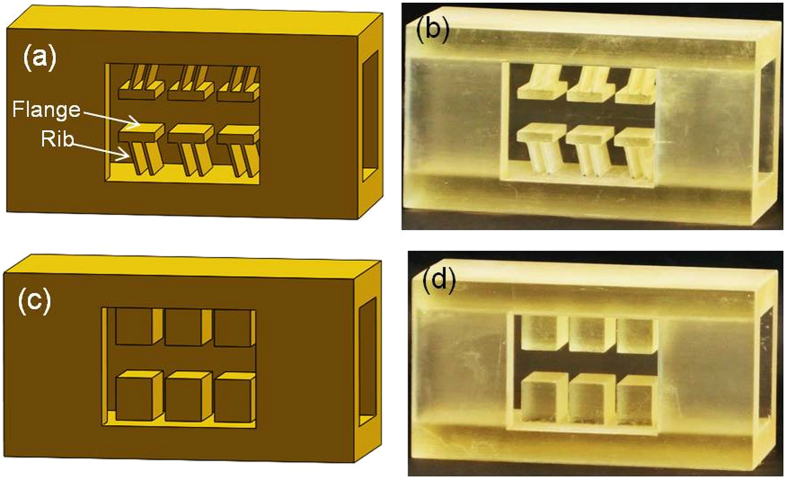
Designed and printed prototype samples. **a,** 3D design of a directionally asymmetric friction IAM with the initial inclination angle of the Z-machine ribs of 75°. This design is referred to as ‘translational rectilinear female IAM’. **b,** 3D printed realisation of the design shown in Fig. a. **c,** 3D design of an IAM with cuboidal blocks exhibiting directionally independent friction. **d,** 3D printed sample with cuboidal blocks.

Based on the principle of directionally asymmetric friction, samples with Z-machines, contrasting those with cuboidal blocks, were designed and fabricated as shown in Fig. 3. The detailed drawings of these devices are presented in Supplementary Fig. S5 online. We note that, unlike in Fig. 1c, in the designs considered therein the features of the inner architecture responsible for directional asymmetry of friction (the ‘ribs’) are imparted on the walls of the IAM, rather than the slider. In the following, we refer to the IAMs with the ribs attached to the slider as ‘male’ and those with ribs attached to the walls as ‘female’.

**Figure 4:**
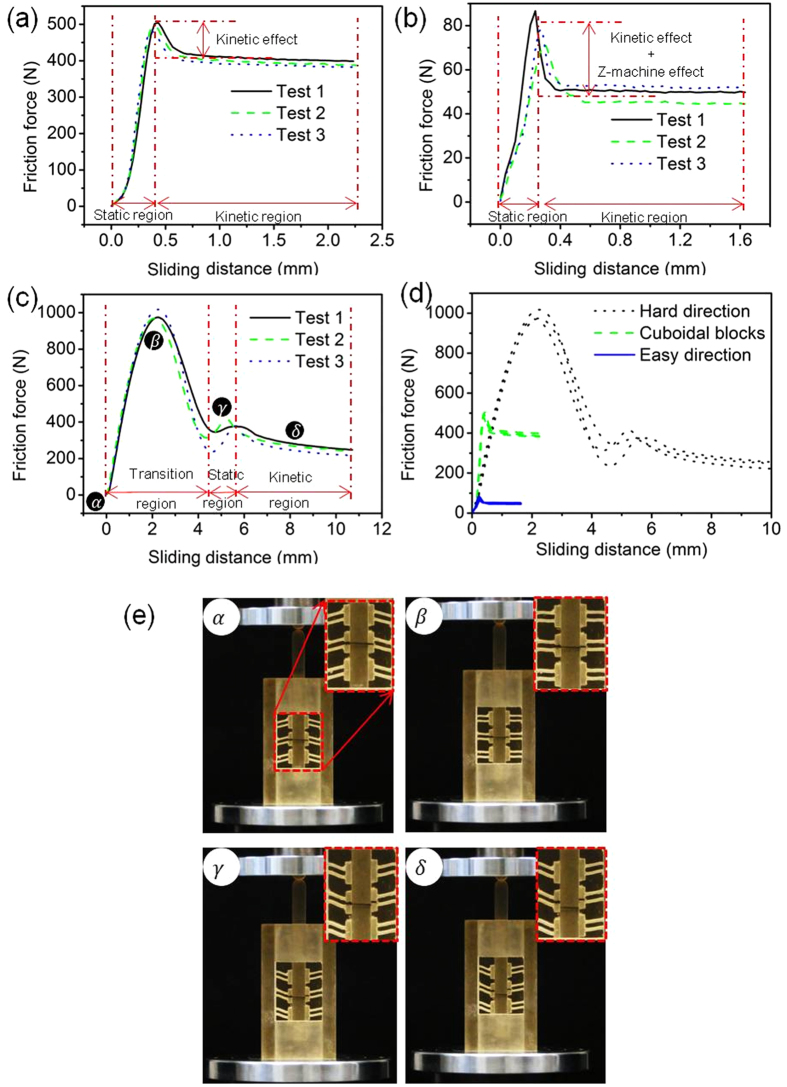
Friction forces in IAMs. **a,** Friction force for a directionally independent IAM with solid cuboidal blocks. **b,** friction force in easy direction for a Z-machine based IAM with directionally asymmetric friction. **c,** friction force in hard direction for a Z-machine based IAM with directionally asymmetric friction. **d,** comparison of friction force in the easy and the hard directions with that for the case of the directionally independent IAM. **e,** deformation of machine ribs at different loading stages labelled in Fig. 4c.

The measured friction forces in the easy and the hard sliding directions for IAMs based on the Z-machine design are shown in Fig. 4, along with the results for a directionally independent IAM with solid cuboidal blocks. While strong directional asymmetry was found for the Z-machine design, the friction force in the case of cuboidal blocks (Fig. 4a) exhibited behaviour similar to the classical friction under a constant normal force (see Supplementary online for the classical friction behaviour). No directional asymmetry was found for the latter case. Rather, the friction force behaviour was similar to that observed in the conventional friction coefficient measurements (cf. Fig. 4a). Two regions can be identified: (i) static friction region where the friction force increased until it reached a peak value around 500 N and (ii) kinetic friction region where the friction force dropped by about 20% from the peak to a plateau level.

The friction force in the easy direction for the directionally asymmetric friction IAM also showed two-region behaviour (cf. Fig. 4b). It should be noted that the static friction force peak decreased from 500 N (in the case of the directionally independent friction IAM) to about 80 N (in the case of the directionally asymmetric friction IAM). This is due to the fact that the force conversion of the Z-machines effectively reduces the normal force during sliding in easy direction, as discussed in Section 2.1. The friction force in the kinetic friction region dropped even stronger, by about 38%.

Figure 4c displays the friction force in the hard direction. In contrast to the friction force in the case of cuboidal blocks or that for the easy direction of the Z-machine based IAMs, three regions can now be distinguished. The first region corresponds to the rotation, or tilting, of the Z-machine ribs with increasing sliding displacement, the rib inclination angle *θ* rising from 75° to 90°; no relative sliding between the bar and the machine flanges is observed in this region. This behaviour is a manifestation of the friction resistance in the hard direction being so high that no sliding is possible. As a result, the ribs (or beams in the model considered in Section 2.2) deform non-linearly (and non-elastically), which gives rise to their rotation.

During the rotation, the friction force increases continuously until it reaches a peak at about 1000 N at 90°. This is followed by buckling of the machine ribs and a decrease of the friction force. It should be noted that after passing the 90° angle, the machine reverses to the ‘easy direction’ configuration as shown in Fig. 4e. Therefore, the decrease in the friction force in this region can be attributed to stress relaxation of machine ribs in buckling and the change in the direction of the normal force as a result of the inclination angle of the machine passing the 90° mark. Essentially, the machine has already failed at this stage.

After the change-over from the hard direction to the easy one, a static and a kinetic regions qualitatively similar to those for the classical friction and easy glide were observed. According to these measurements, this IAM showed a friction asymmetry coefficient of *ξ*=12 – a value that gives us a reason to refer to this asymmetry as a giant one. Figure 4d shows the plots for the friction forces for the directionally independent IAM with solid cuboidal blocks, as well as for the easy and the hard directions in the case of Z-machine augmented IAMs with directionally asymmetric friction property. The results highlight the efficacy of the Z-machines in producing friction asymmetry.

The concept of directionally asymmetric friction can be employed to create other geometrical designs for transitional or rotational movements for various applications. Examples of such designs for five different IAMs and data on their friction asymmetry coefficient are presented in Supplementary online.

### Design of IAMs with Enhanced Friction Asymmetry (Extra High *ξ*-values)

As evident from analysis in previous sections, the main factor affecting the friction asymmetry is the geometry of the Z-machines, which, in turn, controls the force transformation coefficient *F*_*tr*_. This is particularly the case under the conditions close to the infinite sliding resistance case (*μF*_*tr*_*=*1). There are, however, limitations on the magnitude of admissible sliding resistance in the hard direction as it will eventually lead to buckling whereby the machine will be broken. That is why further increase in *ξ*-value can only be achieved by decreasing friction in the easy direction.

However, these conclusions relate to the original design of Z-machines where the ribs had the same bending resistance in both directions. We now explore a special design in which the machine ribs have a directionally dependent bending stiffness, such that bending stiffness in the easy direction is smaller than the one in the hard direction.

**Figure 5:**
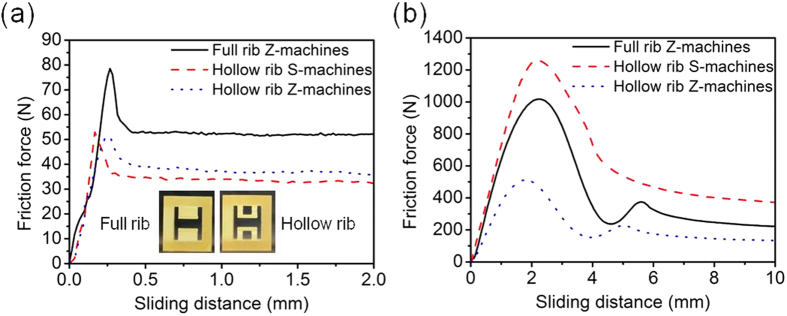
Friction force in IAMs with full rib Z-machines, hollow rib Z-machines, and hollow rib S-machines. **a,** easy direction. **b,** hard direction. The inset photos show side views of the translational rectilinear female IAMs shown in Fig. 3a with full and hollow ribs.

One possible way of achieving directionality of bending stiffness of the ribs is by removing or adding material where appropriate. This can be presented as a two-step process. In a first step, the stiffness of a machine rib (and consequently the sliding resistance force) in easy direction is decreased by removing material from its middle part. The right inset photo in Fig. 5 shows the side view of this design, where it is referred to as ‘hollow rib’, while ‘full rib’ refers to the side view of the original design of translational rectilinear female IAM (Fig. 3a). The sliding resistance forces in easy and hard directions of this design are shown in Fig. 5a,b, respectively. It can be seen that through this change in design the sliding force in the easy direction was reduced from 78 N to 51 N, while in the hard direction the reduction was from 1017 N to 511 N.

**Figure 6:**
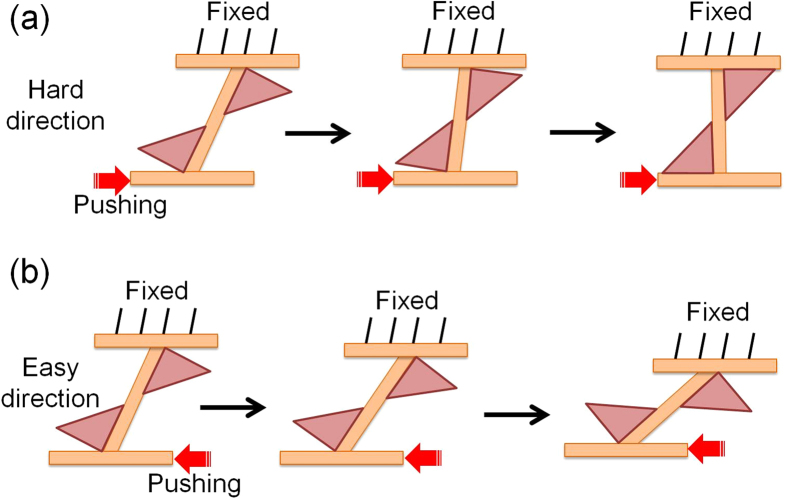
Schematic of an S-machine with directionally dependent stiffness. **a,** pushing the machine rib in hard direction. **b,** pushing the machine rib in easy direction.

A second step consists in adding material to a machine rib to increase its stiffness in the hard direction without influencing that in the easy direction. This is achieved by adding two elements with triangular cross-section to the top and bottom of a machine rib as shown in Fig. 6. When a machine rib is pushed in the hard direction (Fig. 6a), the added triangles develop a contact with the fixed side and the moving flange and start carrying part of the compressive force created due to the tilting of the rib. This, in turn, results in increased bending stiffness and delays buckling and stress relaxation of the rib. By contrast, when the rib is pushed in the easy direction (Fig. 6b), it stays inactive as it has no contact with the flange. Due to a resemblance of such design of the rib with the letter ‘S’, this kind of machine was dubbed *S-machine*.

In order to investigate the directionally dependent stiffness of a rib in the proposed S-machine design, a 2D finite element analysis was performed using commercial ABAQUS software (See Supplementary online for more details). Given a complex geometry of the machines one needs to consider their stability against failure, which is related to possible stress concentrations created by the geometry. In order to characterise the stress concentrations, we will use the equivalent von Mises stress, which is a conventional basis for estimating integrity of structures made of ductile materials, such as the polymers employed in the present 3D printed structures. The von Mises stress distribution in S- and Z-machine ribs in the easy direction showed that the stress is concentrated within a narrow part of the S-machine rib delineated by two parallel dashed red lines. Almost no stresses were observed in the two added triangles (See Fig. S11). However, when the sliding direction was changed to the hard one, the added triangles did contribute to the increased load bearing capability and the resistance to deformation of the ribs. After this design study, prototype IAMs with the newly proposed S-machines were 3D printed. Measurements of the friction force in easy and hard directions were conducted for these prototypes and then compared to the performance of Z-machines with and without a cut in the middle part of the machine ribs, as shown in Fig. 5. The effect of triangle-shaped additions to the ribs turning a Z-machine into an S-machine on the sliding force in easy direction was insignificant, the sliding force increasing from 51 N in the case of the Z-machines with hollow ribs to 53 N. By contrast, the influence of this addition on the sliding force in hard direction was very prominent: it rose from 511 N to 1260 N. Overall, a device augmented with S-machines demonstrated a performance by far superior to that of the original Z-machines and was able to resolve the problems associated with buckling of the ribs (see Fig. S12). We conclude that a change in design from Z- to S-machines provides an IAM or a device in which it is utilized with added asymmetry of the frictional force. The two-step design modification resulted in an increase of the *ξ*-value from around 12 (Z-machine-based design) to a spectacularly high value of about 24 for S-machine based design.

### Application: ‘Mechanical Rectifier’

In electronics, a diode rectifier based on a giant difference in resistance to electric current flow in opposite directions is used to convert alternating current (AC) to direct current (DC). In this work, a directional asymmetry of friction, understood as the difference in the friction resistance to sliding in opposite directions, is used to produce a ‘mechanical diode’ counterpart.

**Figure 7:**
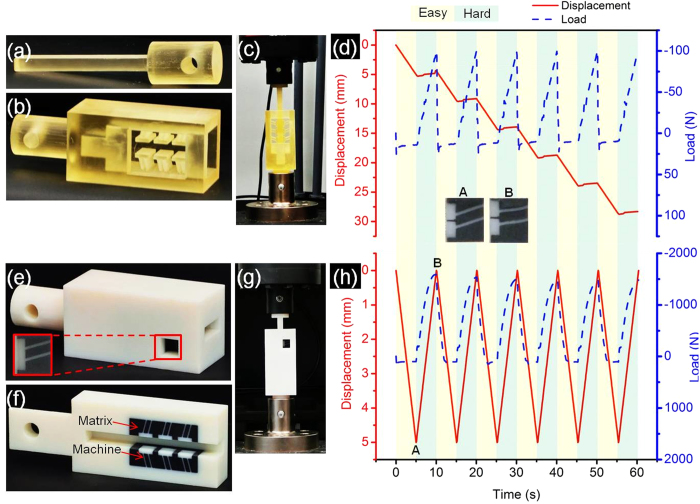
Translational mechanical rectifier. **a,** sliding bar. **b,** rectifier using directionally asymmetric friction IAM without a matrix. **c,** rectifier assembly. **d,** converting an alternating load into unidirectional translational movement. **e,** rectifi**e**r based on a machine augmented composite (the inset photo shows a machine and the surrounding soft matrix). **f,** middle section view of the rectifier. **g,** machine augmented composite rectifier assembly. **h,** alternating movement of the slider in the rectifier with a matrix in high load asymmetry condition. (Inset figures A and B show a Z-machine at the end of the glide in easy and hard direction and the effect of the supporting matrix on the deformation of the ribs).

Currently mechanical rectification is achieved by using various gear mechanisms (e.g.,^31^) or employing impact (commonly bilinear) oscillators (e.g.,^32^). In nature, the most common method of rectification is based on pushing from a firm ground as in walking or hopping (e.g.,^33^), or in robotics, pushing from the ground under vibrations^34^,^35^,^36^. We propose a novel mechanical rectifier that converts alternating loading (with periodically changing load direction) to a translational movement in only one direction, Fig. 7.

To demonstrate the operation of such a rectifier, the following tests were conducted. The sliding bar (Fig. 7a) was connected to the load cell of an Instron machine, while the outer part of a directionally asymmetric friction IAM (Fig. 7b) was affixed to the platform of the machine as shown in Fig. 7c. A cyclic sliding test under a mixed displacement and load control mode was then conducted in the following way. After full insertion of the slider between the flanges of the IAM, a user-defined displacement profile was specified to enable the cross-head of the Instron machine to push the bar downwards in a displacement–control mode. This was done at a speed of 1 mm/s for 5 s. Then, the slider was pulled in a load–control mode until the load reached a set value of 100 N within 5 s. This loading pattern was repeated in each cycle.

The choice of a mixed mode of loading, instead of using displacement control throughout the cyclic test requires an explanation. The pushing step (moving the sliding bar in easy direction) cannot be performed in a load–control regime because the load needed to maintain the movement of the bar in easy direction is higher than the load required for initiating the movement as discussed in Section 2.3 and shown in Fig. 4b. As a result, if the applied load is set to the level required to maintain the movement, sliding will never start. Conversely, if it is set to the level required to initiate the movement, after reaching that level the load will precipitously drop as a result of static to kinetic transition, and then the cross-head of the Instron machine will move at a maximum speed and the displacement control will be lost.

The IAM considered transforms the alternating loading provided by the cross-head movement of the machine into a unidirectional movement, as indicated by the red solid line in Fig. 7d. While the sliding bar started movement in one direction (easy direction) at the peak load of about 25 N, it did not move in the other direction (hard direction) even at a load of about -100 N. This applied force in hard direction was well below the static friction force for this device (1017 N, the peak force in Fig. 4c). Therefore, the movement in hard direction was completely suppressed by the device. As seen from the diagram in Fig. 7, the slider was locked when pulled in the hard direction and moved progressively when pushed in the easy direction. Thus the IAM did act as a linear rectifier or a ‘mechanical diode’.

A limitation of this rectifier is that the applied load in hard direction should not exceed the force required for starting the movement in hard direction, otherwise the Z-machine ribs will buckle as shown previously in Fig. 4e and lose its functionality for the next cycle when sliding in easy direction is effected again. (We note that electric diodes have a similar limitation in terms of voltage.)

Therefore, a mechanical rectifier should be designed to ensure that sliding happens before machine ribs start tilting and buckling when the slider is forced to move in the hard direction. This can be achieved by reducing the coefficient of friction of the materials used in these IAMs, increasing their stiffness, and beefing up the ribs. In addition, the rib inclination angle can be varied to boost the friction asymmetry.

Alternatively, the Z- or S-machines can be embedded in a softer matrix that would fill the empty spaces between them and protect the ribs from buckling. The soft matrix can also assist the machines with returning to their original shape more quickly after being deformed. In the terminology introduced by Hawkins and co-workers^27^,^28^, such a rectifier can be regarded as a machine augmented composite (MAC).

A rectifier of this kind employing an IAM with directionally asymmetric friction was fabricated by multi-material 3D printing (Fig. 7e). A translational male IAM was fully embedded in a soft matrix as shown in Fig. 7f. The contrast in the magnitudes of Young’s modulus of the embedded machines and the matrix was very large (E_Machine_/E_Matrix_ ~ 90). After insertion, the sliding bar (Fig. 7g) was periodically pushed down and pulled up in a displacement-control mode with cross-head speed of 1 mm/s to investigate the rectifying performance of the diode under cyclic loading. A large load asymmetry, with the ratio of the friction resistance force in hard and easy directions of about 15, was observed (Fig. 7h). A weak tendency for a decrease of the peak load in hard direction in Fig. 7h may be attributed to minor degradation of the material with the number of cycles. The inset in Fig. 7h shows ribs during sliding in easy and hard directions and also demonstrates that the matrix prevents complete tilting of the ribs. (This is further illustrated through visualization in Video 1 in Supplementary online). By employing a similar principle, *rotational* rectifiers can be created using rotational IAMs suggested in^29^.

## Discussion

Internally architectured materials (IAMs) with directionally asymmetric friction were proposed based on a soft matrix with embedded Z- or S-machines. These machines involve elements developing a normal force as a response to a tangential force during frictional sliding. We used devices in which a normal stress dependent on the direction of sliding stems from the anisotropy of the material of the slider (or the substrate), with coupling of the shear stress and the normal strain components in a constrained environment. We characterised the degree of friction asymmetry by the ratio *ξ* of the friction force in the hard direction to that in the easy direction and found that depending on the value of the coefficient of friction, there exists an optimal combination of anisotropic compliances that makes the *ξ*-coefficient infinite, thus delivering the highest friction asymmetry. Giant directional asymmetry effects can be realised if one comes close to this critical condition through the choice of the anisotropic compliances.

A particular method of achieving anisotropic coupling and the friction asymmetry is to use a layered (transverse isotropic) material with layers inclined to the direction of sliding. Furthermore, a continuous layered material can be replaced with a set of parallel beams (‘ribs’) inclined to the sliding direction. This general design is akin to an object with bristles, such as a toothbrush head or a cat’s fur. Based on this, we proposed to use Hawkins’ Z-machines to produce IAMs with directionally asymmetric friction. Z-machines of various configurations where manufactured by state-of-the-art 3D printing with polymers and tested with regard to the degree of friction asymmetry. It was shown that Z-machines can give rise to an order of magnitude difference between the friction forces in hard and easy directions. While the ‘cat’s fur’ effect attained with the Z-machine is quite spectacular, a further twofold increase in *ξ* could be achieved by modifying the design of the ribs, which led to S-machines with directionally dependent bending stiffness.

We further showed that directionally asymmetric friction can be used in a new type of mechanical rectifier that converts oscillatory movement into unidirectional movement. The viability of the concept was demonstrated by testing 3D-printed mechanical rectifiers. Possible applications of such IAMs, which we refer to as ‘mechanical diodes’, include locomotion in a constraint environment and energy harvesting from oscillatory noise and vibrations. While in the present work the particular realisations of the frictional IAMs with direction asymmetry were obtained by 3D printing, the underlying concepts are general and can be used for other manufacturing techniques, as well.

## Method

### Fabrication of IAMs using a Multimaterial 3D Printer

Different designs of IAMs with directionally asymmetric friction were prepared in SolidWorks and their CAD files were subsequently employed to produce prototype structures utilizing state-of-the-art 3D printing technology. Prototype single-material samples were fabricated using a Stratasys^®^ Eden 260V and acrylic-based photopolymer materials (FullCure720^®^). Composites (multi-material samples) were built with Stratasys^®^ Connex500 multimaterial 3D printer where the required material stiffness was obtained by blending the stiffest polymer (VeroWhitePlus^®^), which simulates conventional thermoplastics, with the softest one - a rubber-like material TangoBlackPlus^®^. The polymers based on UV-curable urethanes and acrylates offer the possibility of changing their elastic properties over a broad range by varying the mixing ratios of rigid and flexible phases (see Supplementary Fig. S4 online showing a building tray of the 3D printer).

### Measurements of Directionally Dependent Friction Force

Measurements of the friction force for directionally asymmetric friction IAMs (translational rectilinear female IAMs) and for a directionally independent IAM were performed on an Instron 5982 machine in compression mode. The experimental setup consisted of a prototype structure placed on a fixed platform. A solid bar was then inserted between the machine flanges (see Supplementary Fig. S6 online, where the variation of the friction force during insertion of a bar is recorded). Again, we note that in this design it was not the slider, but the walls of the IAM that were architectured to provide directional asymmetry of friction.

**Figure 8:**
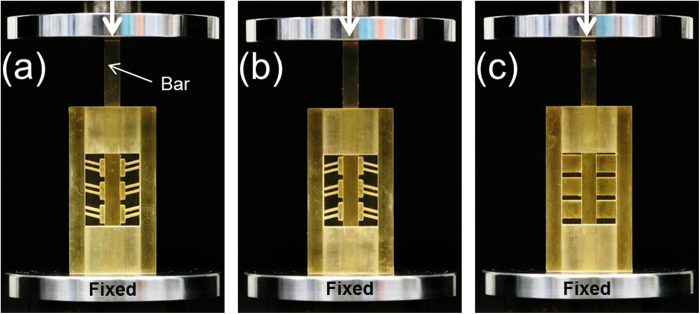
Test setup for IAMs with directionally asymmetric friction. **a,** loading of a translational rectilinear female IAM in easy direction. **b,** loading of a translational rectilinear female IAM in hard direction. **c,** loading of an IAM with cuboidal solid blocks showing no directional asymmetry of friction.

Once the contact between the sliding bar and the Z-machine flanges of the IAM was established, the bar was pushed down with a cross-head speed of 20 mm/min, as shown in Fig. 8. Depending on the orientation of the device, the friction forces in the easy and the hard directions were measured.

## Additional Information

**How to cite this article**: Bafekrpour, E. *et al*. Internally architectured materials with directionally asymmetric friction. *Sci. Rep.* **5**, 10732; doi: 10.1038/srep10732 (2015).

## Supplementary Material

Supplementary Video

Supplementary Information
