# Supplementary material for: Internally architectured materials with directionally asymmetric friction
Source: Sci Rep. 2015 Jun 4;5:10732. doi: 10.1038/srep10732 (PMC4455183; doi:10.1038/srep10732)
Supplement: Supplementary Information [file srep10732-s2.pdf]

Supplementary Information

to the article entitled:

Internally Architectured Materials with Directionally Asymmetric Friction

Ehsan Bafekrpour[1,2], Arcady Dyskin[3], Elena Pasternak[4], Andrey Molotnikov[1,5], Yuri Estrin[1,5]*

[1]*Centre for Advanced Hybrid Materials, Department of Materials Engineering, Monash University, Clayton, Victoria 3800, Australia*

[2]*School of Fashion and Textiles, RMIT University, 25 Dawson Street, Brunswick, 3056, Australia*

[3]*School of Civil and Resource Engineering, The University of Western Australia, 35 Stirling Highway, Crawley, WA 6009, Australia*

[4]*School of Mechanical and Chemical Engineering, The University of Western Australia, 35 Stirling Highway, Crawley, WA 6009, Australia*

[5]*Laboratory of Hybrid Nanostructured Materials, Moscow Institute of Steel and Alloys, Leninsky prosp. 4, Moscow 119049, Russia*

Corresponding author, Email address: yuri.estrin@monash.edu
Tel: (+61) 3 9905 9599, Fax: (+61) 3 9905 4940

This document contains some elements of the theory of directionally asymmetric friction for internally architecture materials (IAMs), as well as experimental details relating to friction tests and manufacturing and testing of IAMs exhibiting friction asymmetry.

Section S1

Calculation of the stress transformation coefficient

The compliance coefficients a_{26} and a_{22} in the Hooke's law, cf. equation (1) of the main text, can be determined for the case of a layered material with the layers inclined to the direction of sliding at an angle θ, as in Fig. 2a, or a Hawkins' Z-machine with an inclination angle of the ribs θ.[1,2]

In the coordinate system (x, y) defined in Fig. 1c, the two-dimensional Hooke's law (plane stress) reads:

$$\begin{cases} \varepsilon'_x = \dfrac{1}{E_l}\sigma'_x - \dfrac{\nu}{E_l}\sigma'_y \\ \varepsilon'_y = -\dfrac{\nu}{E_l}\sigma'_x + \dfrac{1}{E_n}\sigma'_y \\ \gamma'_{xy} = \dfrac{1}{G}\tau'_{xy} \end{cases} \tag{S1}$$

where E_n and E_l are the Young's moduli in the directions normal and parallel to the layers, respectively, G is the shear modulus, and ν is Poisson's ratio.

The corresponding elastic compliances in the coordinate frame (x_1, y_1) can be obtained by rotation of the co-ordinate axes by the angle $-\theta$. The expressions for them read (see, e.g., [3]):

$$a_{22} = \frac{1}{E_l}\sin^4\theta + \left(\frac{-2\nu}{E_l} + \frac{1}{G}\right)\sin^2\theta\cos^2\theta + \frac{1}{E_n}\cos^4\theta$$
$$a_{26} = -\left[\frac{2}{E_n}\cos^2\theta - \frac{2}{E_l}\sin^2\theta - \left(\frac{-2\nu}{E_l} + \frac{1}{G}\right)\left(\cos^2\theta - \sin^2\theta\right)\right]\sin\theta\cos\theta \qquad \text{(S2)}$$

In a special case of a Hawkins' machine, one can use the simplifying assumptions $E_l >> G$, $E_l >> E_n$, and ν~0. Then it follows from equation (S2):

$$F_{tr} = \frac{a_{26}}{a_{22}} = -2\tan\theta \qquad \text{(S3)}$$

This relation appears in the main text as equation (13).

Section S2

Calculation of the force transformation coefficient

We consider a straight beam clamped at one end (point O). The other end is attached to a slider A, which slides over a stationary rigid block B without rotations.

Since the slider cannot rotate, by neglecting the slider dimensions the whole element can be represented by a half of a fully clamped beam of length $2l$ loaded at the middle by concentrated force $2f$, where

$$f = T\sin\theta - N\cos\theta \tag{S4}$$

Deflection of such a beam in local coordinates (z, ζ) shown in Fig. 2a reads (e.g., [4]):

$$\varsigma(z) = \frac{f}{12EI} z^2 \left(3l - 2z\right) \tag{S5}$$

where E is the Young's modulus of the material of the beam, I is the moment of inertia. From here the deflection of the beam end ($z=l$) is $fl^3/(12EI)$. The part Δl of the beam length that would cross the line of sliding is given by

$$\Delta l = \frac{fl^3}{12EI}\cot\theta \tag{S6}$$

The presence of the rigid block has created axial strain in the beam of $\Delta l/l$, which gives rise to an axial force $ES\Delta l/l$, where S is the cross-sectional area of the beam. Then, using equation (S4) and (S5), we obtain the following equation for the normal force N:

$$N = \frac{Sl^2}{12I}\left[T\sin\theta - N\cos\theta\right]\cos\theta \tag{S7}$$

Its solution reads

$$N = T\frac{Sl^2}{12I}\left[1+\frac{Sl^2}{12I}\cos^2\theta\right]^{-1}\sin\theta\cos\theta \qquad \text{(S8)}$$

Finally, the force transformation coefficient is given by

$$F_{tr} = \frac{N}{T} = \frac{Sl^2}{12I}\left[1+\frac{Sl^2}{12I}\cos^2\theta\right]^{-1}\sin\theta\cos\theta \qquad \text{(S9)}$$

The dependence of θ_{max} on the friction angle φ according to equation (15) in the main text is illustrated in Fig. S1.

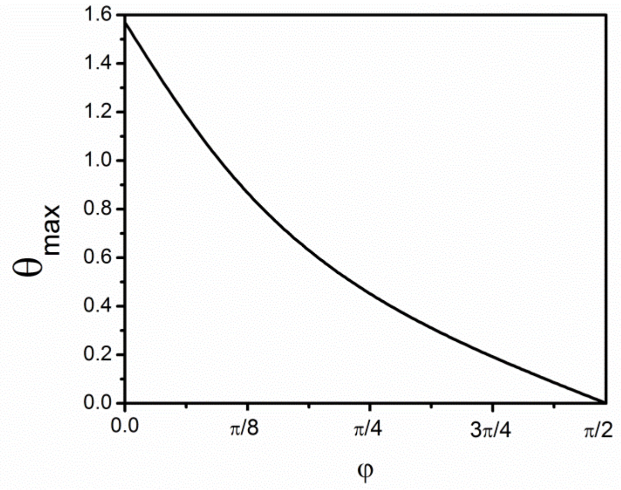

Figure S1. Dependence of the layer inclination angle θ_{max}, which corresponds to a maximum in the friction asymmetry, on the friction angle φ.

Table S1 shows the results of elastic finite element computations of the force transformation coefficient for different values of Poisson's ratio.

Table S1 | The force transformation coefficient for the Z-machine shown in Fig. 2b.

**Poisson's ratio**	**Force transformation coefficient, F_{tr}**
0.10	3.354
0.20	3.350
0.33	3.337
0.44	3.316
0.50	3.299

Section S3

Measurements of the coefficient of friction

As the coefficient of friction is a major factor governing the magnitude of the directional asymmetry of friction, it was measured in a set of experiments on material from which the IAMs were 3D printed. Both the differences in the static and kinetic friction and its dependence on the printing direction, i.e. the direction of movement of the printer nozzles were investigated.

The coefficients of static and kinetic friction were measured according to ASTM D-1894 using a commercially available coefficient of friction tester Model C0008, IDM Instruments Pty Ltd. The experimental setup is depicted in Fig. S2.

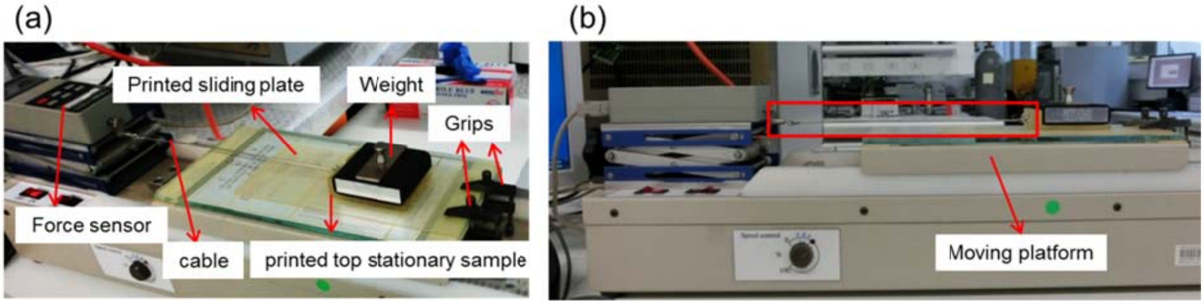

Figure S2 | The friction testing setup. **a**, 3D view. b, front view showing the connector cable is level.

A sled with the contact area of 63.5 mm × 70 mm printed from the same material as the IAMs was placed on a printed plate with dimensions of 100 mm × 220 mm. The sled was stationary and was connected to a force sensor while the plate was mounted on a support platform, which was pulled using a mechanical power unit at a constant speed of 150 mm/min. The pulling force was measured using a Mark-10 digital force sensor. A weight was placed on the top of the sled to provide a normal force of about 2.34 N.

Four different cases were considered:

i. both top and bottom samples were printed with the nozzle moving parallel to the direction of sliding in the friction test, as shown in the inset in Fig. S3a;
ii. the bottom and top samples were printed with the nozzle moving parallel and perpendicular to the sliding direction, respectively, as shown in the inset in Fig. S3b;
iii. the bottom and top samples were printed perpendicular and parallel to the sliding direction, respectively, as shown in the inset in Fig. S3c;
iv. both top and bottom samples were printed with the nozzle moving perpendicular to the sliding direction, as shown in the inset in Fig. S3d.

At least three tests were conducted for each case and the average values were used to calculate the coefficients of static and kinetic friction. The peak force was used to obtain the coefficient of static friction, while the curve after the peak was used to estimate the coefficient of kinetic friction. Fig. S3 shows the experimentally recorded friction forces. The values of the kinetic friction coefficient were determined by averaging over each curve past the peak. Depending on the orientation of printing, the coefficient of static friction varied from 0.31 to 0.37. A smaller degree of variation was recorded for kinetic friction with the coefficient of friction ranging from 0.28 to 0.3. It can be concluded that despite some sensitivity of the friction characteristics to the printing direction, there is sufficient consistency in the results, particularly with respect to the difference between the static and kinetic friction. Hence, representative values for the static (μ_{static} = 0.34) and kinetic ($\mu_{kinetic}$ = 0.29) coefficient of friction were used in finite element simulations in this work.

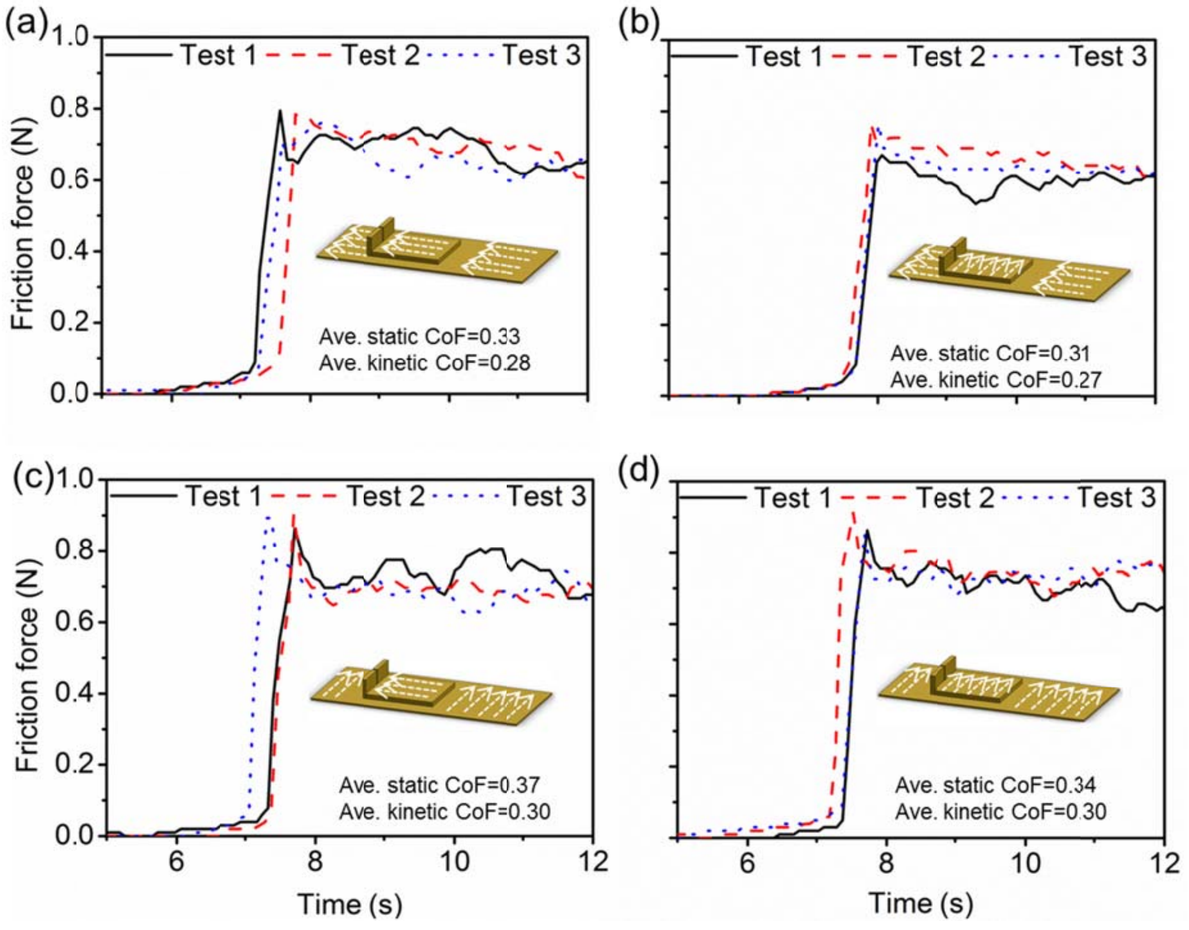

Figure S3 | Friction force vs. time for different printing directions. The inset figures show the top and bottom printed samples for friction test. The dashed arrows indicate the direction of printing. The values of the coefficient of friction (CoF) are shown in the bottom right corner of each diagram.

Section S4

3D-printed IAMs

In this section we present examples of printed IAMs based on Hawkins' Z-machines [1,2] proposed in this article. The terms 'male' and 'female' are used to describe the IAMs fitted with ribs (in the form of Z- or S-machines) attached to the shaft or the walls, respectively.

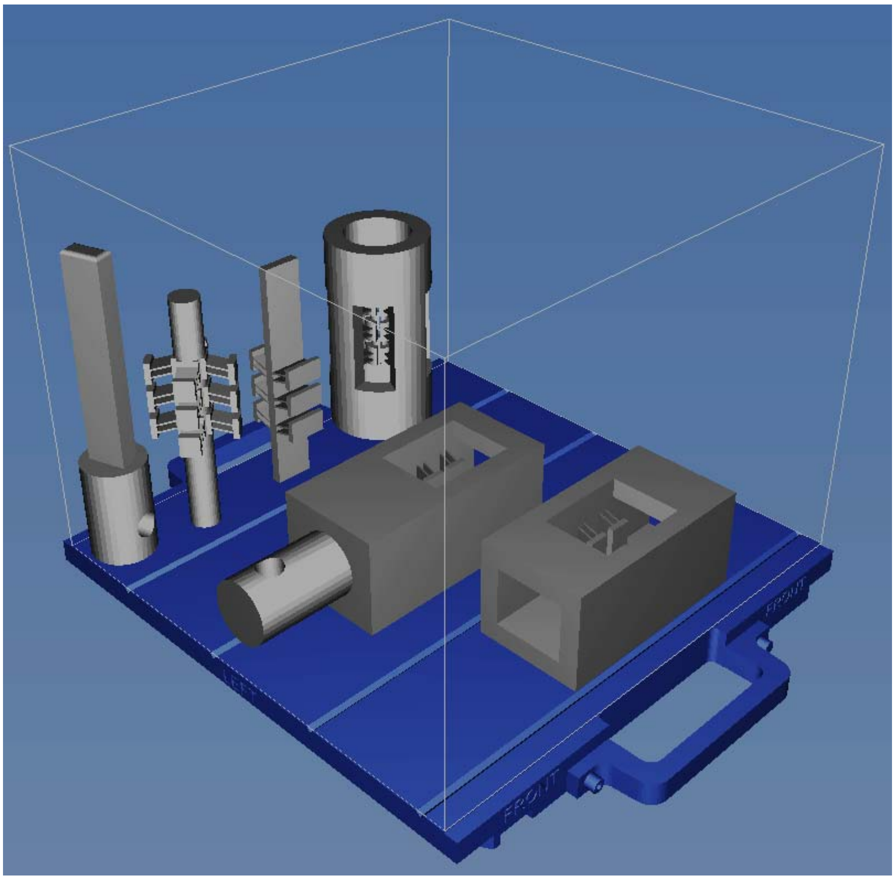

Figure S4 | Orientation of the printed IAMs in the Object Studo: Eden 260V software.

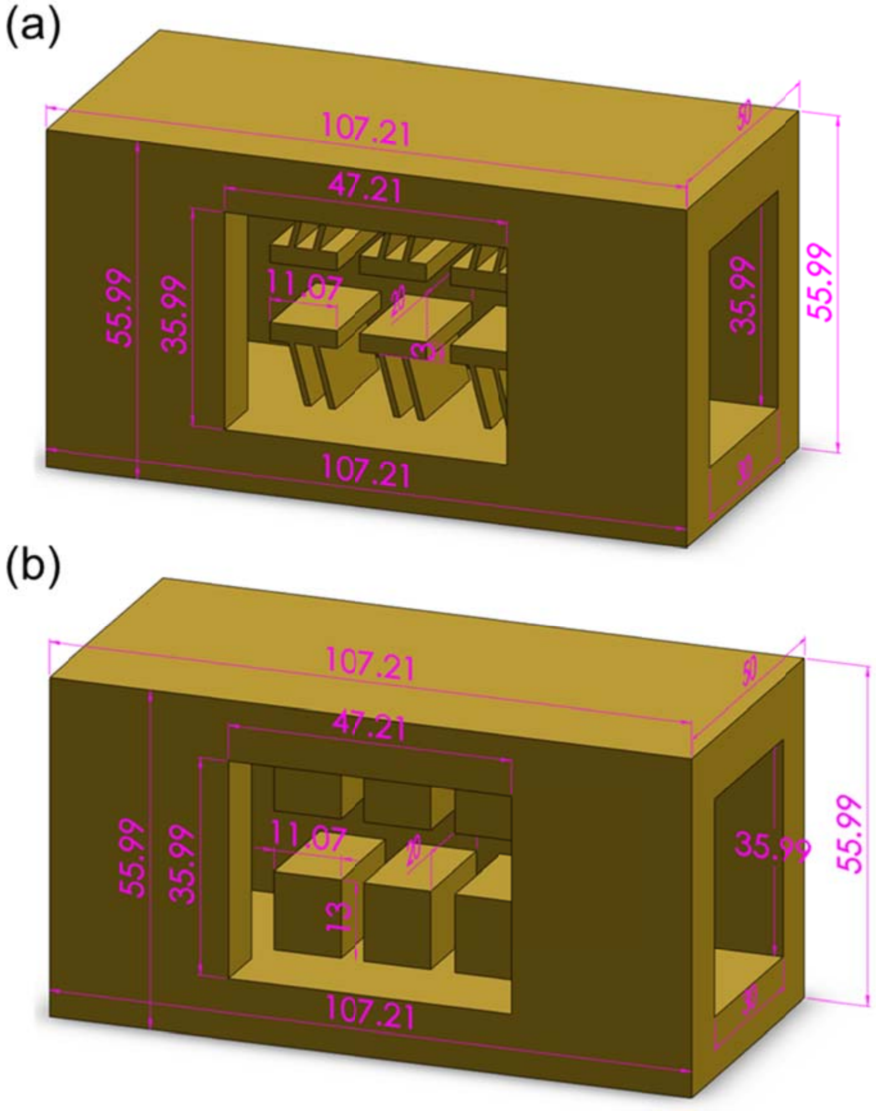

Figure S5. **Dimensions of the samples**. **a**, the translational rectilinear female IAM. b, IAM with solid cuboidal blocks.

Section S5

Variation of the friction force during insertion of a bar in a translational female IAM is shown in Fig. S6. A sharp increase in the friction force at a point when the contact with the next Z-machine was established is noted.

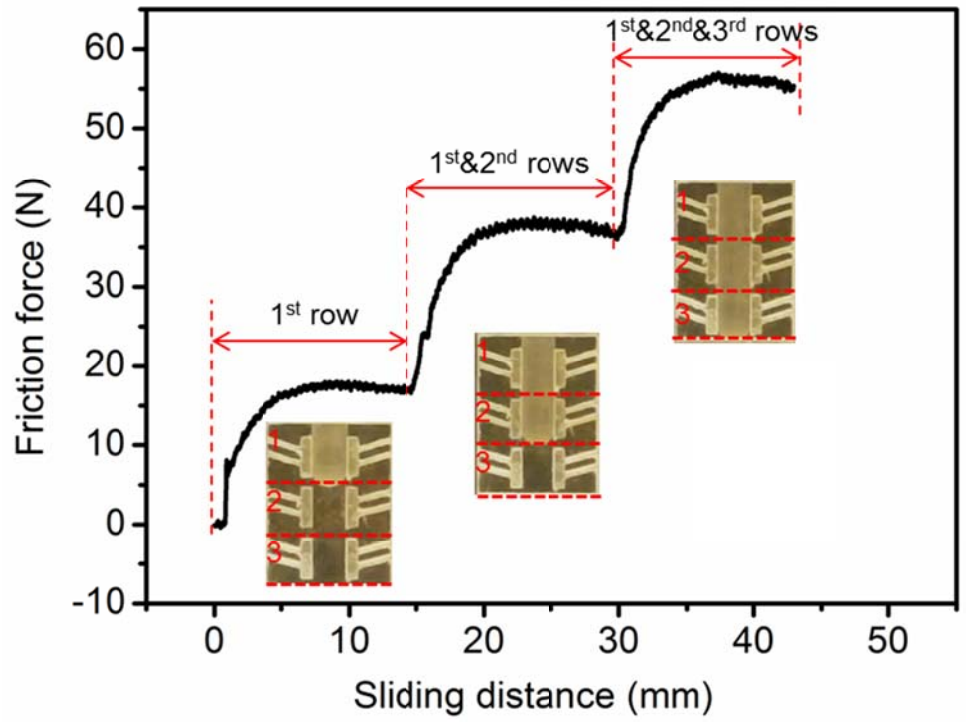

Figure S6 | Variation of the friction force during the insertion of a sliding bar between the Z-machines.

Section S6

Further designs of IAMs realising directionally asymmetric friction

The concept of directionally asymmetric friction can be employed to create other geometrical designs for various applications. In what follows we distinguish between male and female IAMs. As mentioned in the main text, the latter ones are fitted with Z-machines attached to the container with respect to which a smooth slider moves, whilst in the former ones it is the slider that is fitted with Z-machines. Fig. S7a shows a IAMs with directionally asymmetric friction in *translational* movement of a slider with a rectilinear profile. Fig. S7b and S7c display, respectively, the male and female configurations of *circular* IAMs with directionally asymmetric friction in translational movement.

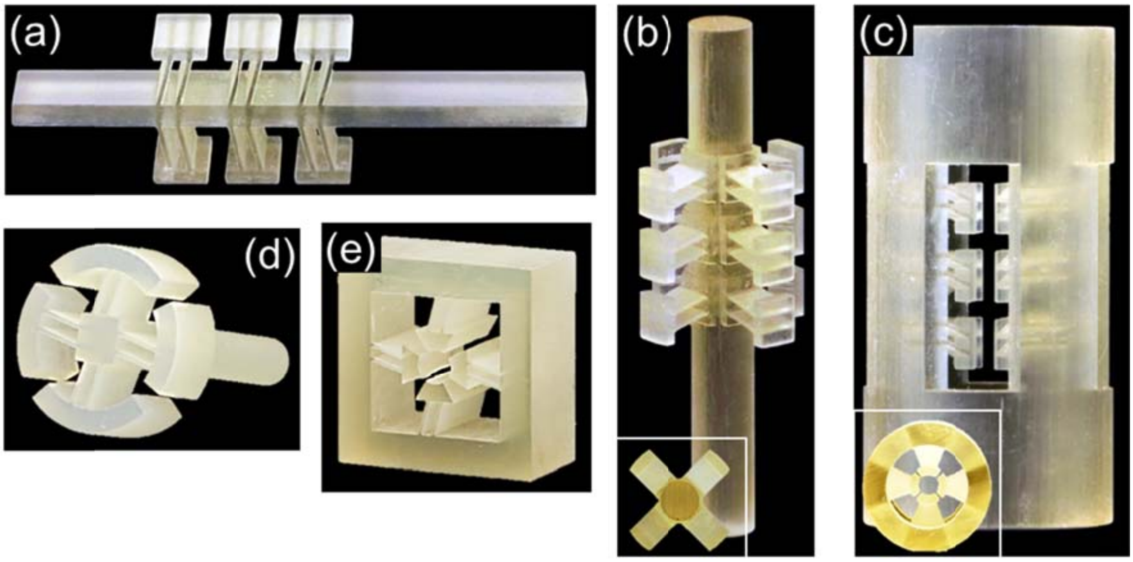

Figure S7 | Further designs of IAMs realising directionally asymmetric friction. **a**, translational rectilinear male IAM. **b**, translational circular male IAM. The inset photo shows a top view. **c**, translational circular female IAM. The inset photo shows a top view. **d**, rotational male IAM, and (e) rotational female IAM.

Based on the same principle, IAMs with directionally asymmetric friction torque in *rotational* movement were also developed, see Fig. S7d and S7e. The sliding force and the torque in easy and hard directions for translational and rotational movements and their respective ξ-values quantifying the magnitude of friction asymmetry are listed in Table S2.

Table S2 | Experimentally measured sliding resistance force or torque in easy and hard directions for translational and rotational sliding, respectively.

	Sliding resistance force (N) or torque (N·m) in easy direction	Sliding resistance force (N) or torque (N·m) in hard direction	Friction asymmetry coefficient, ξ
Model (a)	176 N	1673 N	9.50
Model (b)	232 N	2299 N	9.91
Model (c)	125 N	672 N	5.38
Model (d)	1.2 N·m	4 N·m	3.33
Model (e)	0.55 N·m	1.38 N·m	2.51

Section S7

Experimental test setup for different rotational and translational designs of IAMs

In the case of rotational IAMs with asymmetry of friction depending on the rotation direction, the torque in the easy and the hard directions was measured using a digital torque indicator. The experimental setups for different rotational and translational IAMs are shown in Fig. S8 and S9, respectively.

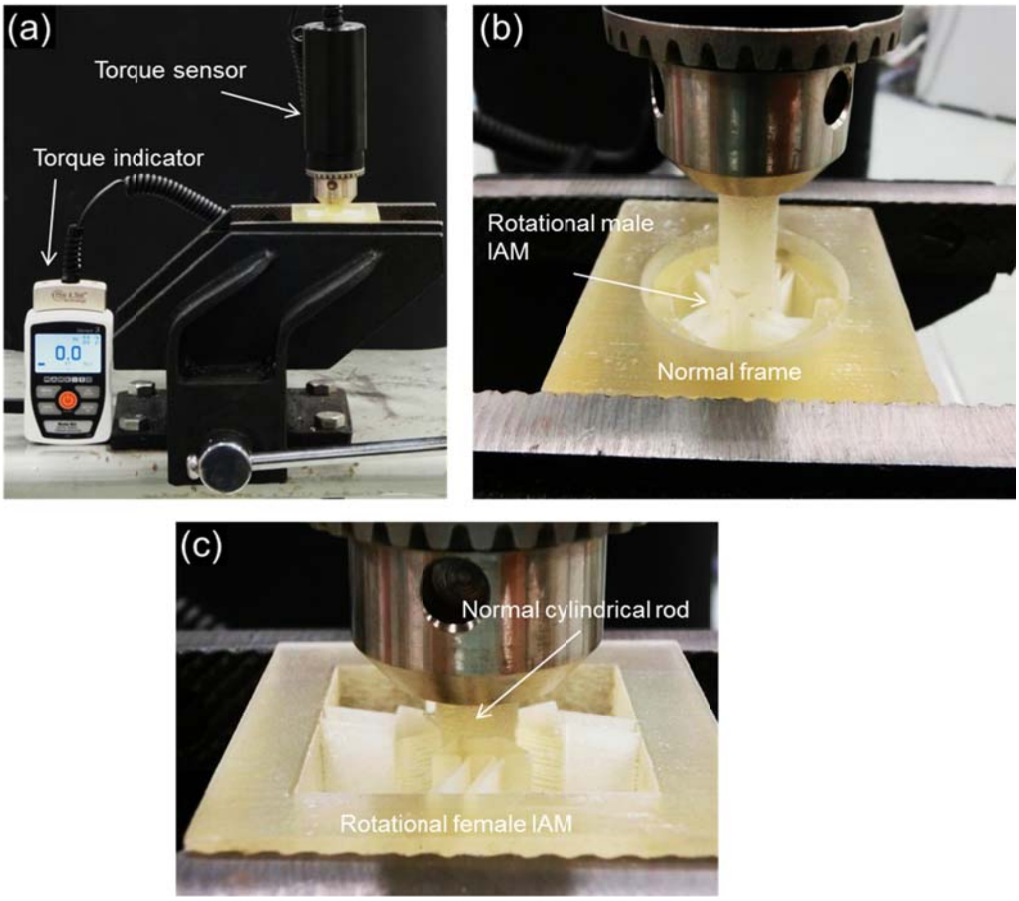

Figure S8 | Test setup for measuring directionally dependent friction torque in rotational IAMs.

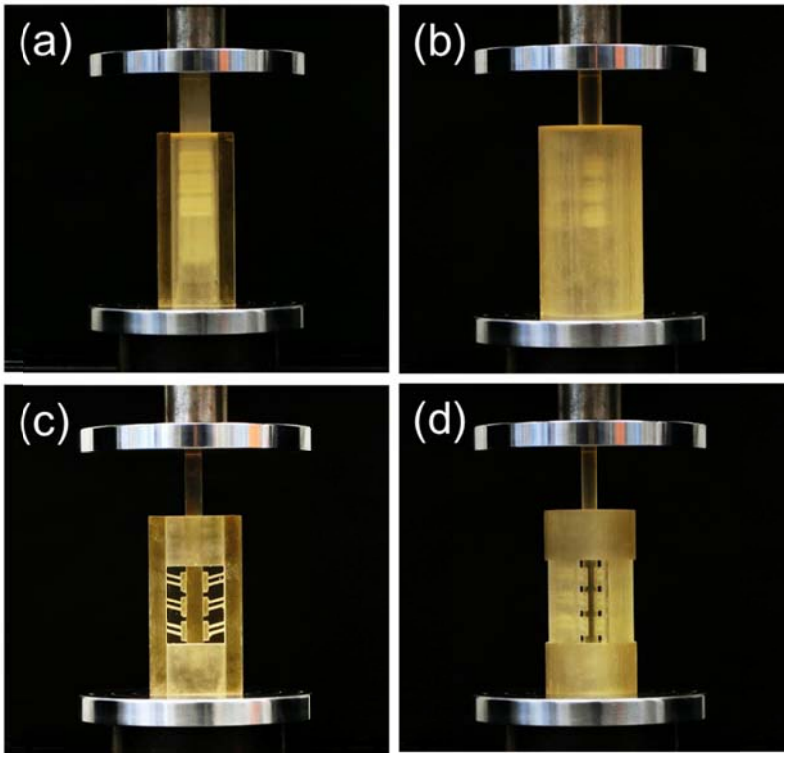

Figure S9 | Experimental test setup for different designs of directionally dependent friction IAMs. **a**, translational rectilinear male IAM. **b**, translational circular male IAM. **c**, translational rectilinear female IAM. **d**, translational circular female IAM.

Section S8

Design and finite element analysis of IAMs with S-machines

IAMs with enhanced asymmetry of friction based on S-machines were introduced in the main text. In order to investigate the directionally dependent stiffness of a rib in the proposed S-machine design, a 2D finite element analysis was performed using commercial ABAQUS software [5]. A representative element of a simulated directionally asymmetric friction IAM consisted of two S-machines with elastic modulus of E=1700 MPa. We used 3-node linear plane stress triangle elements with 0.2 mm in size. The element was pushed in easy and hard directions, as shown in Fig. 6 in the main text. The top flange was fixed and sliding boundary conditions were applied to the bottom flange. The bottom flange was pushed 2 mm to the left for the easy direction and 5 mm to the right for the hard direction. The boundary conditions and dimensions are presented in Fig. S10. Fig. S11 displays distributions of the equivalent von Mises strain in the Z- and S-machines after specified displacements of the respective IAMs in the easy and the hard directions. The deformation of the Z- and S-machines in the printed IAMs after a displacement in the hard direction is seen in Fig. S12.

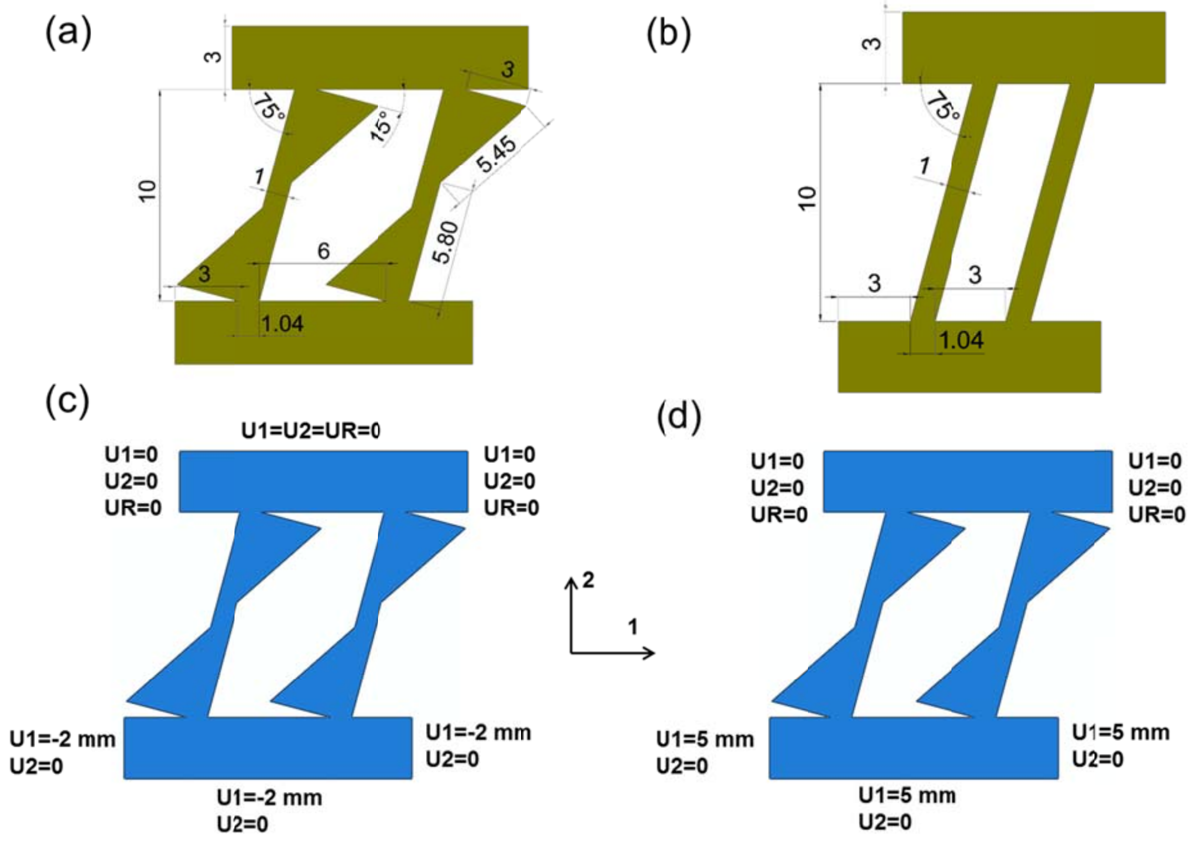

Figure S10. **Dimensions and boundary conditions for the representative S- and Z-machines used in final element simulations with ABAQUS**. **a**, dimensions of an S-machine. **b**, dimensions of a Z-machine. **c**, boundary conditions for easy direction. **d**, boundary conditions for hard direction. The boundary conditions for easy and hard directions, which are the same for both S- and Z- machines, are shown only for an S-machine.

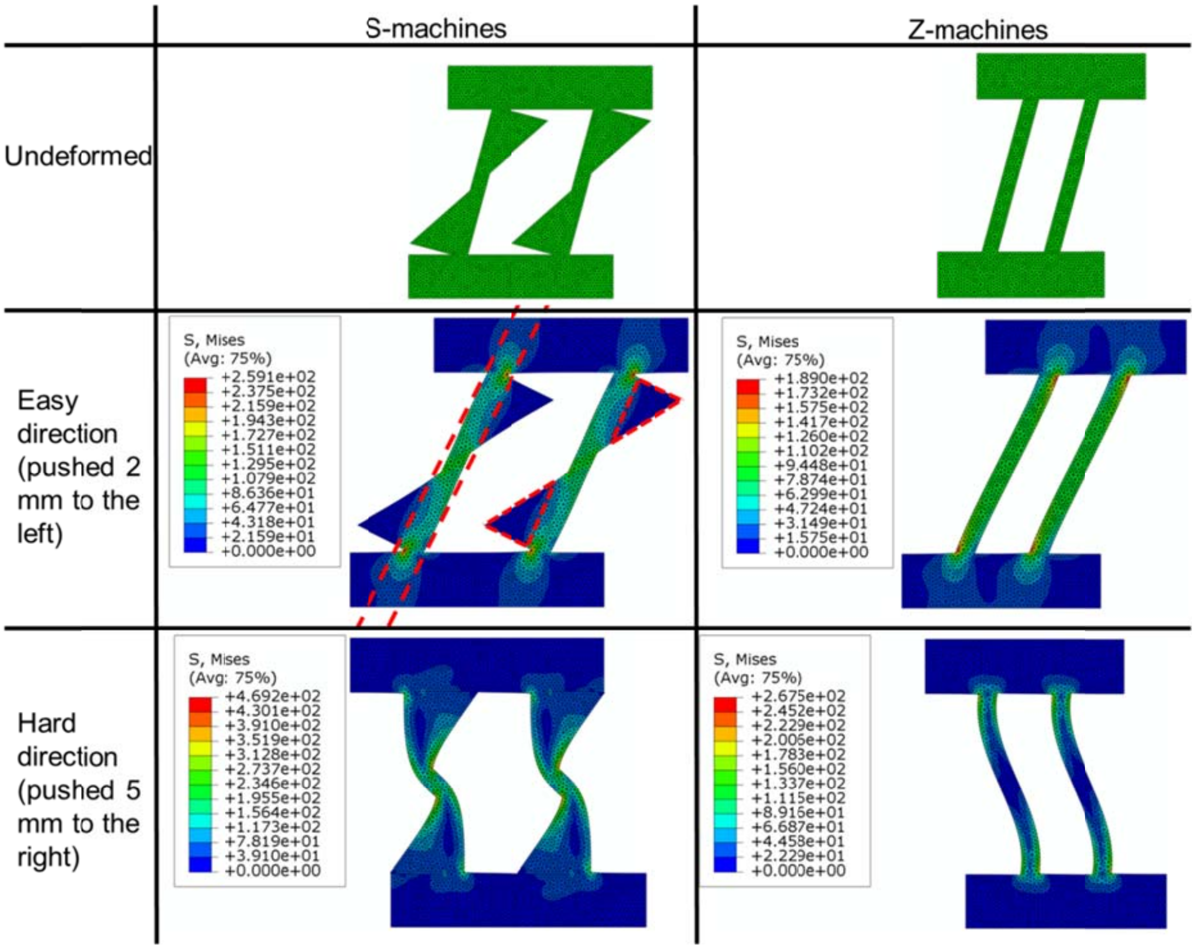

Figure S11. Distribution of the equivalent von Mises stress in S- and Z-machines. The colour code shows the stress levels in MPa.

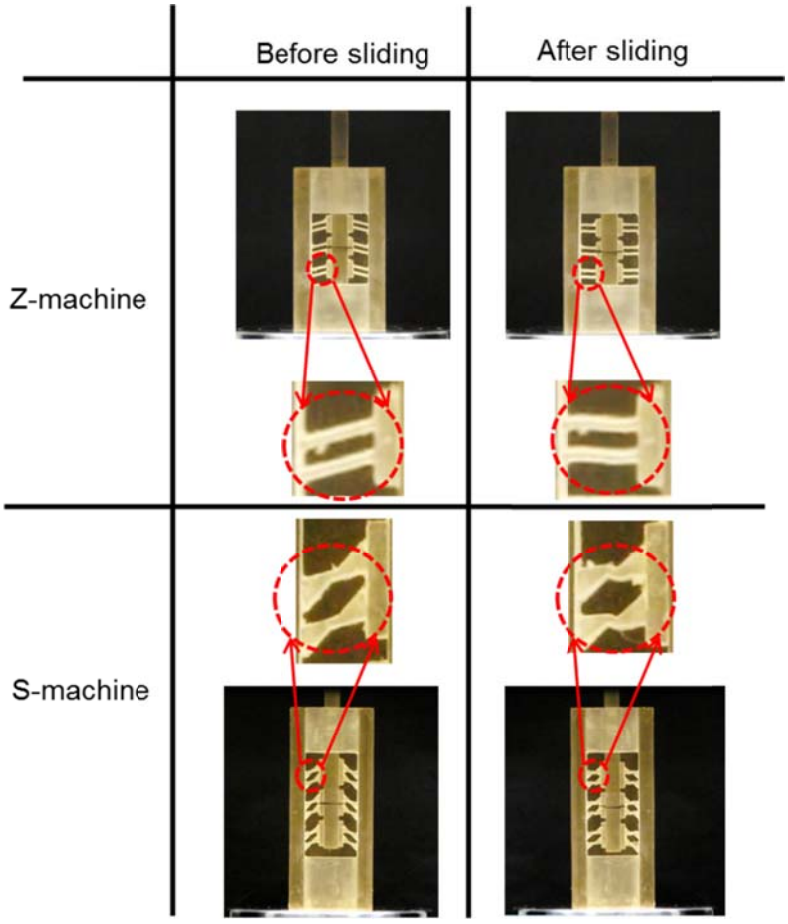

Figure S12. Z- and S-machine based IAMs and deformation of their ribs at the moment when the movement of the slider in the hard direction begins.

References

1. McCutcheon, D. M. *et al.* Damping composite materials by machine augmentation. *J. Sound Vib.* **294,** 828-840 (2006).
2. Hawkins, G. F., O'Brien, M. J. & Tang, C. Y. in *Proceedings of SPIE, Smart Materials III.* 37-45.
3. Lekhnitskii, S. G. *Theory of Elasticity of an Anisotropic Elastic Body*. (Mir Publishers, Moscow, 1981).
4. Landau, L. D. & Lifshitz, E. M. *Theory of Elasticity*. (Pergamon Press, London, 1959).
5. *ABAQUS Documentation and User Manual, Version 6.12, Simulia, Dassault Systèmes*. (2012).
